# Finding a silver lining: the importance of documenting medical tragedies

**DOI:** 10.5195/jmla.2018.408

**Published:** 2018-04-01

**Authors:** Judith A. Wiener

**Affiliations:** Associate Professor and Assistant Director for Outreach and Collections, Health Sciences Library, Ohio State University, Columbus, OH

## Abstract

The radiation overexposure tragedy at a Columbus, Ohio, hospital impacted hundreds of patient lives and made a lasting impression on the regulation and oversight of the use of radiation medicine on a national level. Archival documentation of the incident and the current-day importance of the data collected during and after the event is discussed and highlights many of the reasons why the history of past medical disasters matters to us today.

The Medical Heritage Center at the Ohio State University is typical of many special collections units in health sciences libraries around the globe. Along with the usual medical artifact innovations and oddities, rare medical texts, and personal papers of medical leaders and pioneers, the center also has in its holdings a fair amount of smaller archival collections on a myriad of topics and subjects. These holdings are unique and contain primary sources, unpublished research based on those sources, and firsthand accounts. Hidden in these archival files are often secrets that are unshared or forgotten about and details about how modern medicine came to be and how practice processes became structured and formed.

Quite often in the health sciences, we are imprisoned by the present, overwhelmed by the never-ending stream of information that is being published at an astonishing rate that we rarely take the time to stop and think about why things are the way that they are or how they came to be. Special collections, seemingly far removed from current bench research and not used as a laboratory as they are in the humanities, can be dismissed as frivolous expenses in the modern information network. However, within these collections, there exist the hard-learned lessons of the past that should never be repeated but can also provide useful data that cannot be gotten in any other way but through the study of past tragedies. Such is the case with the Riverside Methodist Hospital radiation disaster.

In March of 1976, a team of neurologists, oncologists, and internal medicine physicians were called together for a meeting with hospital administrators and lawyers at Riverside Methodist Hospital in Columbus, Ohio. The topic of the meeting was to reveal and discuss a grave medical accident that had occurred in treating cancer patients. The incident caused 454 patients who had been treated at the hospital in 1975 and the early part of 1976 to be overexposed to cobalt 60 radiation. The administration originally expressed wishes to keep the overexposure quiet by notifying only living patients who were believed to have received more than 10% of the recommended radiation dose. However, according to George W. Paulson’s unpublished account of the meeting found in the archival files, the doctors vehemently opposed a quiet cover up and immediately demanded that all patients exposed at any level and the press be contacted or they themselves would contact the press. The administration relented and began the notifications that their physicians demanded. The case became known as the largest medical tragedy in the institution’s history and caused changes to radiation treatment and regulations on a national scale.

At the onset of the discovery of the overexposure, the error was blamed on a cracked crystal that inaccurately measured radiation. Yet, as an April 1974 *Columbus Monthly* overview article about the incident written by Linda Stern-Rubin later revealed, the radiation incident was discovered to be a result of human error and lack of oversight [1]. Recommendations, not regulations, once dictated much of radiation medicine, and it was later discovered that the hospital physicist responsible for the equipment had neither checked nor calibrated the radiation therapy equipment since May 1974. It had been recommended at the time and is now required by national agencies that output be measured and calibrated every two to three months.

Qualifications for hospital radiation oversight positions, likewise, were lax in this era. The physicist responsible for the error was young and did not yet have enough experience in the field to qualify for the American Board of Radiology certification. Nonetheless, he was placed as the sole physicist with shockingly limited supervision on several projects and in charge of monitoring the hospital’s radiation equipment. Many, including Rubin, later attributed his failure to calibrate equipment for approximately two years to overextension of his responsibilities, lack of experience, and inappropriate prioritization of his work. When the overexposure was discovered, he first tried to cover up his mistakes by falsifying reports before admitting to his error in judgment and failure to perform routine safety checks.

The result of this medical calamity was multifaceted. As one might fully expect, the reputation and the financial stability of the hospital were placed in great jeopardy as patients began suing the institution. Beyond the financial and legal consequences, a number of patients were affected by the radiation exposure, although, according to Paulson, it is difficult to determine the degree to which many were harmed due to the advanced state of disease that many of the cancer patients already experienced. One physician treating patients at the time later attested that the higher exposure to radiation might have extended some lives or even cured cancers, albeit with a lifetime of radiation side effects.

After the Riverside incident, the *Columbus Dispatch* later reported that the US Nuclear Regulatory Commission conducted a thorough review of all cases and institutional practices and finally created regulations that would close the door to such oversight accidents in the future at all radiation treatment facilities [2]. Perhaps most notably and unexpected, however, was the fact that the accident provided invaluable data for study that would not be available otherwise. Twenty years after the incident, after all the litigation ended, the historical patient data became available for study, resulting in the 1995 journal article, “A Radiation Overdose Incident: Initial Data,” published by three radiation oncologists in the *International Journal of Radiation Oncology, Biology, and Physics* [3]. The data collected during the radiation overexposure have been used to study the radiological effects in a way that could never otherwise be collected by intentional “experimentation” on humans.

In addition to the medical and scientific data gained and regulations that were spurred by this case, lessons were learned in how to best handle such medical situations from an administrative standpoint. One could easily argue that the openness that the Riverside Methodist Hospital physicians insisted upon saved the institution. Today, according to their website, Riverside remains one of the largest hospitals in the central Ohio region and is well regarded for its cardiac, neurology, maternity, and oncology care. What might have meant ruin for the organization instead enabled it to maneuver through a crisis with overall limited subsequent impact. It is a story that has yet to be told from an administrative focus, but one that is also deserving of study in the archival records.

Most history of medicine collection curators can list stories told in their collections similar to this one. A seemingly miraculous new medical discovery is made, only then to be found to have dangerous or even deadly consequences or side effects. Often, regulations, policies, or procedures are put into effect to limit the unintended harm and maximize potential benefit. These stories exist to caution against blindly accepting miraculous discoveries without looking for possible downsides to their unchecked regulation. The records holding such cautionary tales make a difference and can often serve—as time passes, memories fade, and new generations are born—as a collective memory bank. Data gathered during these building years are unique and may never be able to be replicated once limits are known but can be invaluable to future research and discovery.

However, archives can only serve this purpose if they are either sought out or are promoted by dedicated special collections professionals. It is imperative that not only are these materials retained for future study, but that professionals also strive to make them widely available and discoverable. Doing so not only serves to safeguard society against the consequences of tragic misuse of such materials ever again, but also serves to underscore the vital importance of caution in the face of overzealousness, haphazard deregulation, or carelessness in the future.

## References

[1] Stern-Rubin L (1978). The riverside radiation tragedy. Columbus Monthly.

[b2-jmla-106-259] 2Yost M. Regulation could widen to cover radiation error. Columbus Dispatch. Date unknown. Located at: Vertical Subject File Collection. Ohio State University Medical Heritage Center, Columbus, OH.

[3] Cohen L, Schultheiss T, Kennaugh R (1995). A radiation overdose incident: initial data. Int J Radiation Oncology Biol Phys.

